# A spatial analysis of functional outcomes and quality of life outcomes after pediatric injury

**DOI:** 10.1186/s40621-014-0016-1

**Published:** 2014-07-24

**Authors:** Nathaniel Bell, Sami Kruse, Richard K Simons, Mariana Brussoni

**Affiliations:** 1 College of Nursing, University of South Carolina, Columbia, SC USA; 2 Department of Surgery, University of British Columbia, Vancouver, Canada; 3 Department of Pediatrics, University of British Columbia, Vancouver, Canada; 4 School of Population and Public Health, University of British Columbia, Vancouver, Canada; 5 BC Injury Research & Prevention Unit, University of British Columbia, Vancouver, Canada; 6 Trauma Services, Vancouver General Hospital, Vancouver, British Columbia Canada; 7 BC Childrens Hospital, F511, 4480 Oak Street, Vancouver, V6H 3V4 BC Canada

**Keywords:** Wounds and injuries, Spatial analysis, Quality of life, Epidemiology

## Abstract

**Background:**

Changes in health-related quality of life (HRQoL) are more regularly being monitored during the first year after injury. Monitoring changes in HRQoL using spatial cluster analysis can potentially identify concentrations of geographic areas with injury survivors with similar outcomes, thereby improving how interventions are delivered or in how outcomes are evaluated.

**Methods:**

We used a spatial scan statistic designed for oridinal data to test two different spatial cluster analysis of very low, low, high, and very high HRQoL scores. Our study was based on HRQoL scores returned by children treated for injury at British Columbia Children’s Hospital and discharged to the Vancouver Metropolitan Area. Spatial clusters were assessed at 4 time periods – baseline (based on pre-injury health as reported prior to discharge from hospital), and one, four, and twelve months after discharge. Outcome data were measured used the PedsQL™ outcome scale. Outcome values of very low, low, high, and very high HRQoL scores were defined by classifying PedsQL™ scores into quartiles. In the first test, all scores were assessed for clustering without specifying whether the response score was from a baseline or follow-up response. In the second analysis, we built a space-time model to identify whether HRQoL responses could be identified at specific time points.

**Results:**

Among all participants, geographic clustering of response scores were observed globally and at specific time periods. In the purely spatial analysis, five significant clusters of ‘very low’ PedsQL physical and psychosocial health outcomes were identified within geographic zones ranging in size from 1 to 21 km. A space-time analysis of outcomes identified significant clusters of both ‘very low’ and ‘low’ outcomes between survey months within zones ranging in size from 3 to 5 km.

**Conclusion:**

Monitoring patient health outcomes following injury is important for planning and targeting interventions. A common theme in the literature is that future prevention efforts may benefit from identifying those most a risk of developing ongoing problems after injury in effort to target resources to those most in need. Spatial scan statistics are tools that could be applied for identifying concentrations of poor recovery outcomes. By classifying outcomes as a categorical variable, clusters of ‘potentially low’ outcomes can also be mapped, thereby identifying populations whose recovery status may decrease.

**Electronic supplementary material:**

The online version of this article (doi:10.1186/s40621-014-0016-1) contains supplementary material, which is available to authorized users.

## Background

In Canada, an average of 25,500 children between the ages of 0 to 14 are hospitalized annually for injury (SafeKids Canada [2007]). The most common causes of unintentional childhood injuries include: drowning, falls, fires or burns, poisoning, suffocation, and transportation-related injuries (Public Health Agency of Canada [2010]). Injuries sustained in childhood have consequences that can last throughout the life-course, including loss of function, ability, participation, stress and chronic pain (Davey et al. [2005]; Gabbe et al. [2011]). Even children with less severe injuries experience long-term functional impairments and may require long periods of therapy to restore pre-injury health (Polinder et al. [2005]; Rivara [2011]).

Monitoring patient health outcomes following injury is important for planning and targeting interventions. Some studies have shown that behavioral patterns and functional outcomes are modifiable with early intervention (Kenardy et al. [2008]; Johnston et al. [2002]; Gagnon et al. [2009]; Kruesi et al. [1999]). Interventions aid in the recovery from injury and constitute a valuable component to restoring near- and long-term health. However, healthcare resources are often limited, requiring many interventions to be delivered using low cost tools and therapies (Marsac et al. [2011]; Shields et al. [2013]). A common theme in the trauma outcomes literature has been the suggestion that future prevention efforts may benefit from identifying those most at risk of developing ongoing problems after injury and targeting resources and interventions mainly upon them.

Spatial analysis of disease patterns is a robust and low-cost approach for detecting a concentration, or cluster, of outcomes in preparation for targeting prevention and intervention measures (Jerrett et al. [2005]; Musenge et al. [2013]; Cheung et al. [2012]; Takahashi et al. [2008]; MacKinnon et al. [2007]; Green et al. [2003]). Some studies have used spatial cluster analysis to address risk factors associated with injury risk for purposes of prioritizing geographic areas for planning and resource allocation (Warden [2008]; Bell et al. [2008]; Geurts et al. [2005]; LaScala et al. [2000]). There are no such studies demonstrating how these tools can be used to explore geographic distributions of recovery outcomes for purposes of prioritizing post-injury interventions. Spatial analysis could be used to identify unexpected problems in the recovery process and the location of groups of patients at high risk for long-term disability.

In this proof of concept study, we explore the spatial distribution of pediatric injury outcomes within Vancouver, British Columbia, Canada. Our objective was to classify pediatric outcomes into homogenous spatial groups based on self-reported health-related quality of life (HRQoL) responses at different time periods. Public health studies rely on statistical methods for examining determinants that influence the process of recovery after injury. Spatial analysis of recovery patterns may similarly lead to new hypothesis about the contexts that influence the recovery process and how best to target resources to meet patient needs.

## Patients and methods

### Study population

**Figure 1:**
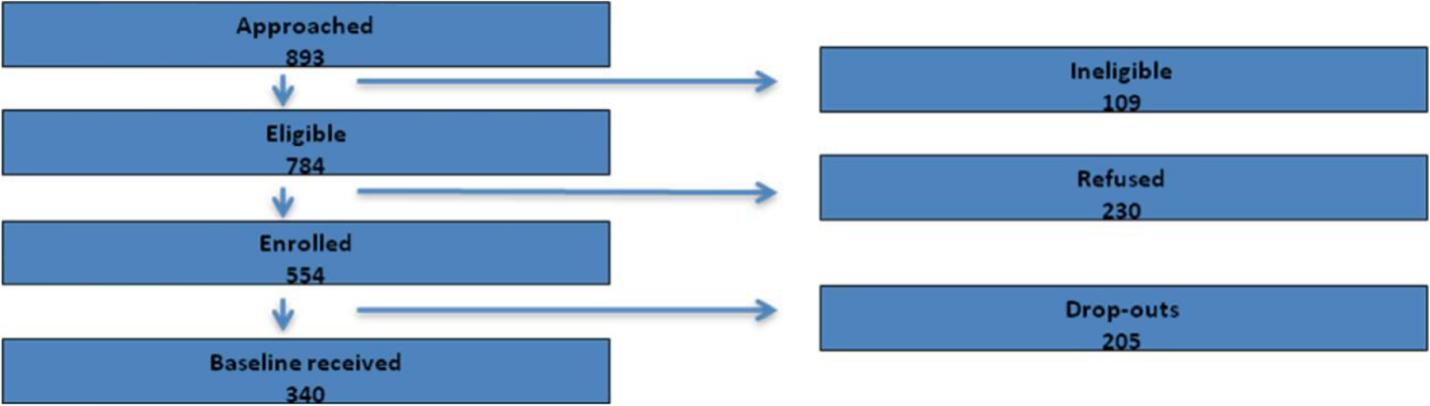
**Study enrollment population.** The spatial scan analysis was constructed using postal code data for participants (n = 154) residing in the Vancouver Metropolitan Area.

Injured children and their caregivers were recruited from the emergency department and in-patient units of British Columbia Children’s Hospital. Patients were eligible for participation if they were seeking treatment for an injury, were aged 0–16, resided within the province and had working knowledge of the English language. A research assistant gave all participants verbal and written explanation of the study. Caregivers provided written consent for participation and children ages seven and over also provided assent for participation. The study recruitment ran from February 2011 to December 2012. During this time 340 of the 784 persons who were eligible for the study completed a baseline interview. Figure 1 illustrates the study consent and enrolment cohort. The analysis presented here is based on baseline and follow-up data among persons whose postal codes were within the Vancouver Metropolitan Area (n = 154).

### Instrument

The PedsQL™ 4.0 Generic Core (Varni et al. [2001]) and the PedsQL™ Infant Scales (Varni et al. [2011]) were used to collect health-related quality of life (HRQoL) data for children ages 2–16 years and 0–24 months. Both measures support the theoretical framework that HRQoL is a multidimensional construct and includes physical, emotional, cognitive and social health dimensions (Varni et al. [2011]; Varni et al. [2003]). The PedsQL™ 4.0 Generic Core consists of 23 items and four subscales: physical functioning, emotional functioning, social functioning and school functioning. The Infant Scales are composed of 45 items and five subscales: physical functioning, physical symptoms, emotional functioning, social functioning and cognitive functioning. A five point Likert response scale ranging from “never” to “almost always” was used in both instruments to assess how different items may affect the child. For both measures, individual item scores were obtained by reverse scoring items and linearly transforming them to a scale of 0 to100.

Psychosocial and Physical Health Summary Scores were computed to allow for stratified analyses of HRQoL outcomes. For the Generic Core, the Physical Health Summary Score is identical to the physical functioning subscale while the Psychosocial Summary Scale is presented as the sum of the emotional, social and school functioning scales and divided by the number of items answered (Varni et al. [2003]). The Infant Scales Physical Health Summary Score includes both the physical functioning and physical symptoms scales and the Psychosocial Summary Score combines the emotional, social and cognitive functioning scales (Varni et al. [2011]).

### Data collection

Participation involved completing a questionnaire package in hospital, and at one, four, and twelve months post-injury. For follow-up, participants were mailed a paper copy of the questionnaire with a self addressed and stamped envelope and also provided with a web link and given the option to reply online. The PedsQL™ instrument, included in each questionnaire, asked the participant to consider their health state during the past one month when responding to questions. As such, baseline participants’ responses reflect their health status before the injury as reported prior to their initial discharge from hospital. The entire survey questionnaire also asked participants to describe the time and place of the injury event, as well as whether the individual had any disability, long-term health problems, or prior injury preceding to the injury event. Each person was also asked to complete a posttraumatic stress disorder questionnaire, provide information about household income, as well as describe the nature injury, the body part affected, and the injury intent.

### Spatial scan statistic

The spatial scan statistic is a method for measuring spatial clustering of event data within adjacent geographic areas and over closely overlapping time periods (Naus [1965]). Kuldroff’s spatial scan statistic is a widely used spatial cluster analysis method (Kulldorff [1997]). It is both a deterministic model, in that it identifies the locations of clustering, and an inferential model, in that it allows for the evaluation of significance of each cluster (Chen et al. [2008]). The method can be used to calculate spatial, temporal, or spatiotemporal clustering of events (Kulldorff et al. [2005]).

Kuldorff’s spatial scan statistic identifies clusters using a scanning window placed at different spatial coordinates within the study area. The scanning window can be defined in kilometers and can take the shape of a circle, ellipsoid, or other shapes. Scan statistics are based on likelihood ratio tests. The most likely cluster(s) are identified from comparing the number of events contained within the scanning window against the maximum value of the likelihood ratio test statistic. The null hypothesis of the scan statistic is that events contained within each scanning window are randomly distributed in geographic space. The alternative hypothesis is that within the scanning window there is an increased risk of events as compared to the risk of events outside of the window. Different probability models are used for spatial scan statistics depending on the nature of the data. Under different models the expected number of events within each window can be measured using only counts of events, or in proportion to a background risk; for example, census population counts or emergency department volume.

### Data analysis

We applied Kuldorff’s scan statistic for ordinal data (Jung et al. [2007]). In some clinical studies, the primary outcome variable is the change in a patient’s condition after treatment. This is often measured on a Likert outcome scale as follows: 1 = much improved, 2 = slightly improved, 3 = no change, 4 = slightly worse, 5 = much worse. Similar scales are used to quantify socio-economic status, cancer stages, and self-rated health responses. In this study, PedsQL™ responses were transformed into quartiles representing different classifications of HRQoL states for each time period relative to the other responses from the survey. The first through fourth quartiles were coded as follows: 1 = very low HRQoL, 2 = low HRQoL, 3 = high HRQoL, and 4 = very high HRQoL, with scores of 1 representative of the worst outcome and scores of 4 representative of the best outcome. The scan statistic for ordinal data detects clusters for all four health states, thus producing a detailed assessment of HRQoL response scores that could represent very low, low, high, or very high HRQoL responses. In this way, the clusters do not always represent the ‘worst’ responses (e.g. 75^th^ – 100^th^ percentile), but could also represent clusters of persons whose health may likely deteriorate (e.g. 50^th^ – 75^th^ quartile) or those whose health outcomes are substantially better than others (e.g. 1^st^ – 25^th^ percentile).

All respondent data were aggregated into Census Tract (CT) administrative geographic boundaries. This was accomplished first by linking the respondents postal code of residence to the Statistics Canada Postal Code Conversion File (PCCF) geographic linkage dataset (Statistics Canada [2009]) and then by linking the PCCF to the CT data file. CT’s are small and relatively stable geographic areas with a population of 2,500 to 8,000 and roughly correspond in size to an urban neighbourhood. In total, 410 CT areal units within the Vancouver Metropolitan Area were used in the analysis. The spatial scan statistics were derived using the geographic centroid for each CT. Centroids were calculated using a commercial geographic information system (GIS) software package.

Candidate clusters were identified from the CT centroids that fell within the scanning window. The radius of the window was programmed to vary continuously, with a maximum search area not exceeding 50% of the population at risk. A maximum of 50% was used because once the scanning window covers more than half the geographic region and/or time period, the likelihood no longer reflects a cluster of increased risk inside the scanning window (Kulldorff et al. [1998]). Each cluster was evaluated using Monte Carlo hypothesis testing on 999 random replications of the data. All areas with a likelihood ratio exceeding 95% of those obtained from the simulation were considered statistically significant. Results were mapped using relative risk ratios for each CT centroid.

We constructed two spatial cluster analyses using participant HRQoL responses. The first analysis was a purely spatial cluster analysis. In this model, the spatial clusters were identified using all HRQoL scores irrespective of the time period when they were provided. In other words, the clusters were representative of quartile scores of those areas that produced similar scores at any point in time (e.g. pre-injury or in follow-up). Thus, a significant cluster could be identified from an area that was in the lowest quartile at time period 1 (baseline) and the lowest quartile at time period 3 (4 months), but not at time period 2 or 4, and so on. This model represents an aggregate assessment of HRQoL over the entire study period. Significant spatial clusters are those areas that consistently produced high- or low HRQoL responses irrespective of the time period when the responses were recorded.

The second analysis was a space-time cluster analysis. In this analysis, observed events in a cluster at each time period were compared to what would be expected if both the spatial and temporal locations of all events were independent of each other. Time periods were assigned as follows: 0 = baseline, 1 = month one, 2 = month four, and 3 = month twelve. In contrast to the spatial analysis model described above, any cluster that was identified using this method was representative of geographic areas having individuals reporting similar HRQoL responses during the same time period. Both the purely spatial and space-time models were constructed using only event (i.e. count) data, but each event was labeled using its quartile ranking. In this way, events could be analyzed with respect to its value.

Table 1 summarizes the nine data fields generated from the cluster analysis to aid in the interpretation of the results. For interpretation of Tables 2, 3, 4 and 5 it is important to note that data values within the columns ‘categories’, ‘observed’, ‘expected’, and ‘RR’ are all referring to the same set of observations. Thus, the numerical value of the first data character in the ‘categories’ column is similarly represented as the first numerical value in the remaining columns. For example, in cluster #3 in Table 2, the ‘categories’ column specifies that the cluster is singularly represented by HRQoL scores 1 (very low HRQoL), 2–3 (low HRQoL and high HRQoL), and 4 (very high HRQoL). HRQoL response scores 2 and 3 are combined since there are not so many counts of events with either a score of 2 or a score of 3 relative to the other scores. Reading from left to right, one can identify that within cluster #3 there are 12 observed counts of events having scores equal to 1; 75 instances with scores equal to 2 or 3, and 49 instances of scores equal to 4. The expected number of events for each score within the geographic area of its size was 32.5, 63.8, and 39.7, with the resulting rate ratio also provided.

**Table 1:** **Summary of the data fields generated by the spatial scan statistic for categorical HRQoL data**

Field	Name	Summary
1	Cluster	Unique identifier assigned to each cluster. ID is re-generated for each cluster analysis.
2	Categories	HRQoL response categoriy groupings determined from the data. Category 1 = very low HRQoL, 2 = low HRQoL, 3 = high HRQoL, 4 = very high HRQoL. Although the purpose of the spatial scan statistic is to detect clusters with high rates or low rates of specific outcomes, this does not necessarily mean that the detected clusters will also produce an outcome pattern in a linearly high or low manner. For example, it is possible for a cluster to be significant with a high rate of low HRQoL scores (value = 1) compared to HRQoL scores of 2, 3, or 4. It is also possible for a cluster to contain a significantly high concentration of scores equal to 1 compared to scores of 2 and 3 combined.
3	Observed	The number of instances an HRQoL response category was observed.
4	Expected	The number of instances an HRQoL response category was expected. Expected observations derived from the size of the scanning window.
5	RR	Relative risk (RR) attributed to each data category. RR scores provide a convenient single number summary of the direction and magnitude of the HRQoL groupings. Areas that generate statistically significant RR scores greater than one map the geographic locations where poor (or conversely, good) patient outcomes have clustered whereas areas that generate statistically significant RR scores less than one map those areas where there is a decreased risk of either a good or poor outcome.
6	p-value	The statistical significance of the spatial cluster (95%)
7	CTs in cluster	The number of census tracts (CTs) contained within the cluster. Counts of populations within each CT can be obtained by summarizing the scores generated in field 3.
8	Radius (km)	The search radius of the scanning window.
9	Time Period	The space-time scan statistic indicates at what time period the spatial cluster was evident.

**Table 2:** **Purely spatial analysis of PedsQL** **™** **physical health summary scores over the entire study period**

Cluster	Categories	Observed	Expected	RR	p-value	CTs in cluster	Radius (km)	Time period
1	[1, 2, 3, 4]	[13, 9, 2, 0]	[5.6, 5.7, 2.4, 10.4]	[2.4, 1.6, 0.8, 0.0]	0.003	7	1.4	…
2	[1, 2-3, 4]	[16,3,2]	[4.9, 7.0, 9.1]	[3.5, 0.4, 0.2]	0.005	36	5.4	…
3	[1, 2-3, 4]	[10, 3, 0]	[3.0, 4.3, 5.6]	[3.4, 0.7, 0.0]	0.042	9	2.7	…
4	[1, 2-3, 4]	[4, 24, 41]	[16.2, 23.0, 29.8]	[0.2, 1.1, 1.4]	0.168	24	2.8	…
5	[1-3, 4]	[0, 9]	[5.1, 3.9]	[0.0, 2.3]	0.457	3	3.1	…
13	[1-3, 4]	[0, 6]	[3.4, 2.6]	[0.0, 2.3]	0.992	1	0.0	…

Categories: 1 = very low, 2 = low, 3 = high, 4 = very high.

**Table 3:** **Purely spatial analysis of PedsQL**™ **psychosocial health summary scores over the entire study period**

Cluster	Categories	Observed	Expected	RR	p-value	CTs in cluster	Radius (km)	Time period
1	[1, 2-3, 4]	[95, 105, 59]	[62.0, 121.5, 75.6]	[2.0, 0.8, 0.7]	0.004	76	14.3	…
2	[1, 2, 3, 4]	[5, 14, 27, 36]	[19.6, 20.1, 18.4, 23.9]	[0.2, 0.7, 1.5, 1.5]	0.007	81	21.0	…
3	[1, 2-4]	[12, 75, 49]	[32.5, 63.8, 39.7]	[0.3, 1.2, 1.3]	0.035	29	3.7	…
4	[1, 2, 3, 4]	[7, 0]	[1.7, 5.3]	[4.3, 0.0]	0.110	4	1.3	…
5	[1, 2, 3, 4]	[0.0, 0.7, 1.3, 1.8]	[6.7, 6.9, 6.3, 8.2]	[0.0, 0.7, 1.3, 1.9]	0.140	4	1.2	…
11	[1, 2-3, 4]	[6, 4, 0]	2.4, 4.7, 2.9]	[2.6, 0.9, 0.0]	0.997	8	2.6	…

Categories: 1 = very low, 2 = low, 3 = high, 4 = very high.

**Table 4:** **Space-time analysis of PedsQL** **™** **physical health summary scores over the entire study period**

Cluster	Categories	Observed	Expected	RR	p-value	CTs in cluster	Radius (km)	Time period
1	[1, 2-3, 4]	[37, 20, 13]	[16.4, 23.4, 30.3]	[2.5, 0.9, 0.4]	0.001	66	4.5	1 to 1
2	[1, 2-4]	[9, 0]	[2.1, 6.9]	[4.4, 0.0]	0.013	17	2.9	1 to 2
3	[1, 2-3, 4]	[8, 0]	[1.9, 6.1]	[4.4, 0.0]	0.049	12	4.7	1 to 2
4	[1, 2-4]	[9, 1, 0]	[2.3, 2.3, 5.3]	[4.0, 0.4, 0.0]	0.058	17	4.8	1 to 1
5	[1, 2, 3, 4]	[13, 1, 0]	[6.6, 1.4, 6.1]	[2.0, 0.7, 0.0]	0.490	23	4.8	1 to 2
8	[1, 2-3, 4]	[0, 8]	[4.5, 3.5]	[0.0, 2.3]	0.975	5	1.6	2 to 3

Categories: 1 = very low, 2 = low, 3 = high, 4 = very high.

Time period: 0 = baseline, 1 = month one, 2 = month four, 3 = month twelve.

**Table 5:** **Space-time analysis of PedsQL** **™** **psychosocial health summary scores over the entire study period**

Cluster	Categories	Observed	Expected	RR	p-value	CTs in cluster	Radius (km)	Time period
1	[1-2, 3-4]	[15, 0]	[7.3, 7.7]	[2.1, 0.0]	0.064	22	3.9	0 to 1
2	[1, 2, 3, 4]	[10, 8, 1, 0]	[4.5, 4.7, 4.3, 5.5]	[2.3, 1.7, 0.2, 0.0]	0.066	17	2.3	2 to 3
3	[1-2, 3, 4]	[0, 6, 9]	[7.3, 3.4, 4.4]	[0.0, 1.8, 2.1]	0.152	30	4.2	0 to 1
4	[1,2-3, 4]	[2,26,24]	[12.4, 24.4, 15.2]	[0.2, 1.1, 1.6]	0.278	12	2.6	0 to 1
5	[1, 2-3, 4]	[13,9,1]	[5.5, 10.8, 6.7]	[2.5, 0.8, 0.2]	0.746	15	1.3	0 to 1
14	[1, 2-4]	[4, 0]	[1.0, 3.0]	[4.2, 0.0]	0.999	3	1.3	0 to 1

Categories: 1 = very low, 2 = low, 3 = high, 4 = very high.

Time period: 0 = baseline, 1 = month one, 2 = month four, 3 = month twelve.

## Results

**Figure 2:**
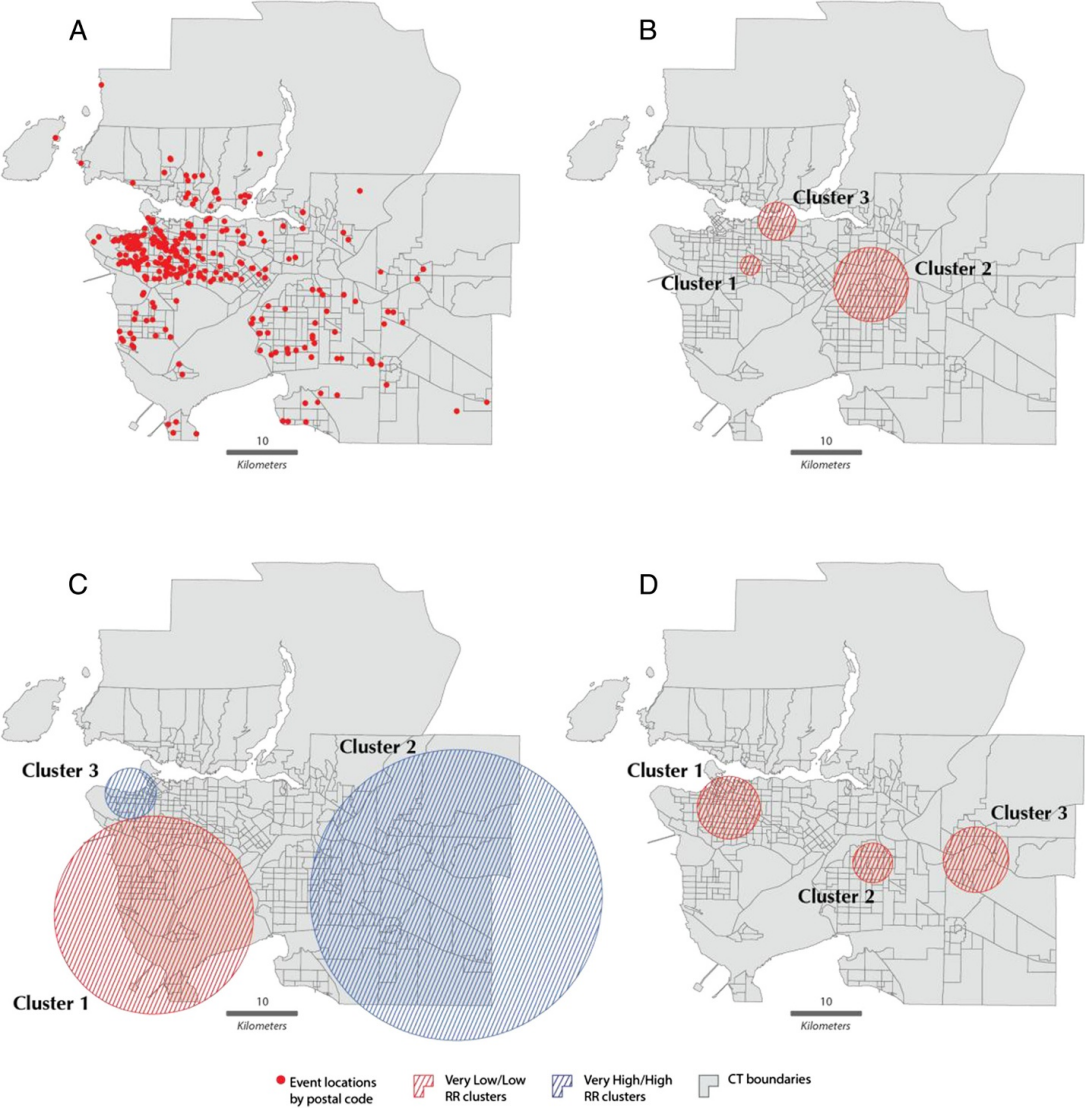
**Spatial cluster analysis of PedsQL health summary scores across Metropolitan Vancouver.** **(A)** Geographic locations of respondent data by six digit postal code. All respondent surveys were aggregated into their corresponding Census Tracts prior to running the analysis; **(B)** Purely spatial scan statistic showing only statistically significant clusters based on PedsQL™ physical health summary score over the entire study period; **(C)** Purely spatial scan statistic showing only statistically significant clusters based on PedsQL™ psychosocial health summary score over the entire study period; **(D)** Space-time statistic showing statistically significant clusters based on PedsQL™ physical health summary scores over specific locations and time periods.

The analysis is based on 154 physical and psychological responses to the PedsQL™ survey at baseline (pre-injury), 1 month, 4 months, and 12 months after injury. All responses were included in the analysis irrespective of whether an individual completed all or only a portion of the follow-up surveys. For the physical health component of the PedsQL™, 30 individuals elected to complete only the first assessment (pre-health survey), 17 completed the first two assessments, 47 the first three assessments, and 60 completed all four assessments. For the psychological health component of the PedsQL™, 28 individuals completed only the first assessment, 17 completed the first two assessments, 47 the first three assessments, and 60 completed all four assessments. There were no statistically significant differences in demographic or injury-related characteristics of participants who elected to participate for the full year versus those who dropped out or re-enrolled (Table 6). The residential postal codes used to build the spatial cluster model are shown in Figure 2A.

**Table 6:** **Characteristics of the 154 participants by full and partial participation**

	Withdrew/Re-Enrolled (n = 91)	Full participation (n = 60)	p-value
Age (SD)	8.0 (4.8)	9.0 (4.7)	0.22
Sex			0.71
Male	63.7	66.7	
Female	36.3	33.3	
Total household income			0.38
< $14, 999	1.2	0.0	
$15,000 - 29,999	3.5	5.3	
$30,000 - 59,999	22.1	10.5	
$60,000 - 79,999	14.0	14.0	
> $80,000	59.3	70.2	
Hospitalized	13.2	6.7	0.11

All values percentages unless otherwise noted.

Comparison p-values based on independent t-test for participation age, chi-square test for household income and sex, and Fisher's exact test for Hospitalization due to small cell size.

In model 1, the purely spatial analysis, statistically significant clusters of HRQoL response scores were identified within 3 of the 13 clusters of PedsQL™ physical health summary scores. The results are displayed in Table 2 and in Figure 2B. Clusters 1 through 3 contained concentrations of very low and low HRQoL scores (categories 1 and 2) relative to good recovery scores. The geographic concentration of these clusters ranged from 1.4 to 5.4 kilometers, containing 52 CTs in total. Clusters 4 and 5 contained areas with concentrations of good recovery scores (Category 4), however these clusters were not statistically significant in comparison to other response patterns within the same area (clusters with p values > 0.05 are not mapped).

Statistically significant clusters were identified within 3 of the 11 clusters of psychosocial summary scores. The results are displayed in Table 3 and Figure 2C. Cluster 1 contained concentrations of poor HRQoL scores in comparison to all other responses whereas clusters 2 and 3 contained concentrations of good recovery scores in comparison to all other responses. The geographic distance of these clusters ranged from 3.7 to 21.0 kilometers, with the concentration of poor scores contained within 76 CTs and the concentration of good recovery scores contained in 110 CTs.

In model 2, the space-time analysis, statistically significant clusters scores were identified within 3 of the 8 clusters generated from the PedsQL™ physical health summary scores. The results are displayed in Table 4 and in Figure 2D. Cluster 1 contained concentrations of very low scores in comparison to all other responses during time period 1; the first month post-injury. Clusters 2 and 3 contained concentrations of areas with respondents reporting very low HRQoL scores relative to all other responses across time periods 1 and 2; months one and four post-injury in addition to a concentration of populations reporting high and low scores. The cluster radius ranged from 2.9 to 4.8 kilometers, containing 95 CTs in total. No statistically significant clusters of HRQoL response scores were identified from the psychosocial summary scores when measured over space and by follow-up period. Spatial scan statistics for the space-time analysis of psychosocial summary scores are shown in Table 5.

## Discussion

In this study, a methodology was presented using pediatric PedsQL™ responses that can be applied for identifying spatial concentrations of populations reporting similar functional outcome and health-related quality of life scores after injury. Three observations can be constructed from the results. First, the data provide initial evidence that injury outcomes cluster geographically. Second, the data show that geographic concentrations of similarly reported health-related quality of life scores are identifiable at specific time periods after injury. Lastly, the results suggest that geographic areas with significant concentrations of good or poor physical health summary scores are not necessarily aligned with the geographic concentrations of good or poor psychosocial health recovery.

One potential application of the spatial scan statistic is to identify concentrations of geographic areas where populations report similar outcome scores. Geographic locations identified from this methodology may be the first place to target interventions at different time periods after discharge since they contain significant concentrations of patients classified into similar risk categories. Similarly, this methodology also identifies concentrations of potentially at-risk areas (e.g. areas with concentrations of ‘low’ HRQoL responses), which is important for identifying those who are potentially at-risk for experiencing poorer outcomes over time. Conversely, identifying clusters of good recovery outcomes is relevant for identifying factors that materialize geographically at the local or neighborhood scale that are beneficial for recovery.

Public health surveillance requires statistical analysis of outcome data for examining determinants that influence the process of recovery from injury. However, most statistical tests produce global results, providing little information about local variations in outcomes. Given limited resources, health care providers must be strategic for targeting surveillance and interventions in order to maximize positive impacts. Spatial scan statistics are tools to identify *where* specific outcomes occur, thereby providing initial information about specific groups patients at high risk for long-term disability. Spatial scan statistics for ordinal data categorize outcomes, thereby providing information not only on the most extreme outcomes, but also about those who are likely to ‘fall through the cracks’ if their health were to deteriorate.

Despite its significance as a leading public health problem for children, data on injuries are very limited in Canada among pediatric populations. Most often, researchers must rely on administrative data sets that were not designed for injury surveillance purposes. No center systematically collects data on post-injury outcomes, which means our understanding of the recovery process after hospitalization and the services that may be needed to support recovery is virtually non-existent. In our view, a prospective assessment of geographic patterns of injury outcomes is important to (1) identify trends and patters in changing needs, (2) evaluate whether policy and practice changes implemented as a result of these types of analyses are actually having the intended effect, and (3) for identifying whether there are sub-populations that need specific support.

The methodology proposed in this study is exactly what is needed to determine where there may be need for more post-injury support and for developing new hypothesis about the recovery process. For example, if data were routinely available from individuals over time, such information could be used to (a) plan the order in which populations would receive treatment; (b) deliver interventions based on areas that have the greatest overlap with other areas; (c) determine if areas with significant concentrations of poor outcomes contain significant concentrations of populations with low resource utilization; or (d) determine if interventions delivered to specific areas resulted in a reduction or elimination of health.

With regards to objective (a), interventionists could visit sites in a way that might maximize time or maximize fuel or resource consumption. With regards to objective (b), outreach programs could be initiated at locations that were equidistant between two neighboring areas, thereby resulting in the greatest likelihood that populations from both communities would attend. With regards to objective (c), an overlap analysis between clusters of low or very low outcomes with clusters of low or very low resource utilization statistics (e.g. outpatient rehabilitation visits) could lead to the identification of populations who may benefit from alternative therapies, such as telemedicine rehabilitation modalities. Use (d) could be evaluated by determining whether an intervention resulted in significant changes in functional outcomes or HRQoL after controlling for SES both overall as well as within the specified geographic area. However, this would require additional vigilance to the individual-level data that were controlled for in the analysis. For example, certain factors (e.g. high school education) are less likely to be indicative of change in socio-economic position after injury relative to other factors (e.g. change in one-year income). Potentially more interesting, however, would be to use the spatial scan statistic to help determine ‘what is the geographic extent that communities remain influential determinants of health and well-being after injury?’ For example, previous studies have shown that certain features from the built environment, such level of cohesion, crime rates, employment opportunities or better access to transportation, as are important determinants of recovery following injury.(Liang et al. [2008]; Hagglund et al. [2009]) Such studies could benefit from quantifying just how far the impact of ‘community’ spans, thereby helping to determine either the buffer zone whereby these meso-level factors cease to remain influential on health outcomes, and thereby the locations in which additional interventions/resources could be targeted.

Some limitations of this analysis should be recognized. First, this study was limited to a relatively small cohort of trauma patients with the majority of persons requiring less than 24 h stay in hospital. Moving forward, future applications of spatial scan statistics on trauma outcomes would benefit from stratifying outcomes by injury grade (e.g. ISS 0 – 8, 9 – 15, > 15) or mechanism to identify whether geographic clustering of outcomes changes by severity or mechanism. Secondly, we did not exclude response data from participants who withdrew from the study at different intervals. The analysis was on the collective pattern of recovery within geographic areas. Therefore, the results should not be interpreted as a spatial analysis of individual outcomes. However, with population-based registries individual-based surveillance could be feasible. Thirdly, we conducted this analysis without adjusting for known covariates associated with recovery, nor did we present on all the different types of scan statistics that have potential application in trauma outcome studies. Continued study can improve our understanding of how injury severity, mechanism, demographics, socio-economic, or environmental factors influence the spatial distribution of outcomes. With regards to the third limitation, one additional spatial scan statistic that warrants further exploration in the analysis of geographic clustering of outcomes post-trauma is the Poisson permutation model. (Kulldorff [1997]) For example, the Poisson permutation model can adjust for multiple covariates within each cluster, thereby allowing practitioners to quantify how much impact in outcomes or in health services utilization is determined through geographic location. Lastly, as our primary objective was to identify concentrations of both ‘high risk’ and ‘potentially high risk’ populations based on physical and psychosocial survey responses, we measured observed and expected cases based on numerator data. Future assessments whereby risk was based on counts of specific outcomes (e.g. number of visitations to physiotherapy, days since discharge) could account for the background population at risk relative to the number of events within each geographic area. It should also be noted that our rationale for including only those persons who resided within the Vancouver Metropolitan Area was multifaceted. For example, the nature of Census Tracts limited the analysis to urban areas as CT’s are only produced for metropolitan areas with a base population of at least 50,000. It was also necessary to ensure anonymity in response data, which could not be maintained for rural areas do to the small numbers.

## Conclusion

In this study of pediatric physical and psychosocial outcomes we demonstrated the utility of a spatial scan statistic for identifying significant clusters of high- and low-risk areas of a poor recovery after injury. Ordinal spatial scan statistics identify different clusters of risk categories, thus producing a detailed assessment of clustering across the different health states. Identifying these clusters is relevant to targeting prevention interventions based on need. This methodology could be integrated into a tiered response protocol, whereby hospitals with limited budgets could target intervention groups by geographic area and by need. Continued development and exploratory analysis of spatial scan statistics will further refine possible strategies of these tools for monitoring patient outcomes after injury. These results provide initial evidence that outcomes post-injury similarly result in defined geographic clusters and also add to the trauma outcomes literature by introducing a methodology for exploring spatial clustering of outcomes over space and over different time periods during recovery.

## Authors’ original submitted files for images

Below are the links to the authors’ original submitted files for images. Authors’ original file for figure 1 Authors’ original file for figure 2
