# Supplementary figures and images for: A spatial analysis of functional outcomes and quality of life outcomes after pediatric injury
Source: Inj Epidemiol. 2014 Jul 24;1(1):16. doi: 10.1186/s40621-014-0016-1 (PMC4648946; doi:10.1186/s40621-014-0016-1)

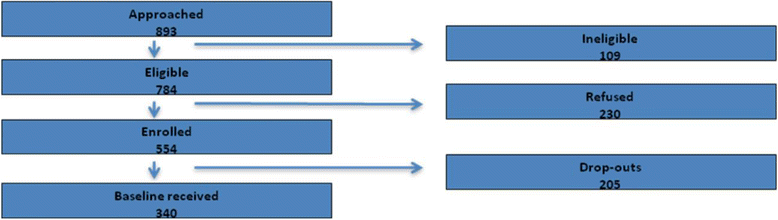

Supplement: Supplementary file 1 — Authors’ original file for figure 1 [file 40621_2014_16_MOESM1_ESM.gif]

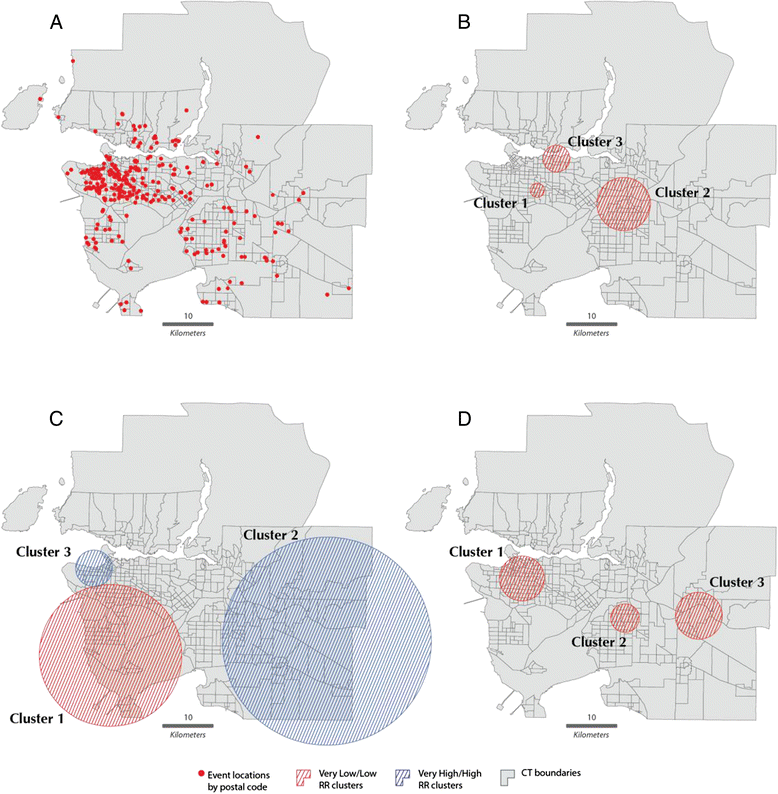

Supplement: Supplementary file 2 — Authors’ original file for figure 2 [file 40621_2014_16_MOESM2_ESM.gif]
